# New immunological potential markers for triple negative breast cancer: IL18R1, CD53, TRIM, Jaw1, LTB, PTPRCAP

**DOI:** 10.1007/s12672-021-00401-0

**Published:** 2021-03-10

**Authors:** Paolo Marchetti, Alexey Antonov, Lucia Anemona, Chaitania Vangapandou, Manuela Montanaro, Andrea Botticelli, Alessandro Mauriello, Gerry Melino, M. Valeria Catani

**Affiliations:** 1 Oncology Unit, Department of Clinical and Molecular Medicine, University of Rome La Sapienza, 00185 Rome, Italy; 2 MRC Toxicology Unit, University of Cambridge, Cambridge, CB2 1QR UK; 3 Department of Experimental Medicine, Torvergata Oncoscience Research (TOR), University of Rome Tor Vergata, via Montpellier 1, 00133 Rome, Italy

**Keywords:** Triple negative breast cancer, IL18R1, CD53, TRIM, Jaw1, LTB, PTPRCAP, Prognostic markers, Precision oncology, Cancer immunity

## Abstract

Breast cancer (BC) is the second leading cause of cancer death in women worldwide, and settings of specific prognostic factors and efficacious therapies are made difficult by phenotypic heterogeneity of BC subtypes. Therefore, there is a current urgent need to define novel predictive genetic predictors that may be useful for stratifying patients with distinct prognostic outcomes. Here, we looked for novel molecular signatures for triple negative breast cancers (TNBCs). By a bioinformatic approach, we identified a panel of genes, whose expression was positively correlated with disease-free survival in TNBC patients, namely IL18R1, CD53, TRIM, Jaw1, LTB, and PTPRCAP, showing specific immune expression profiles linked to survival prediction; most of these genes are indeed expressed in immune cells and are required for productive lymphocyte activation. According to our hypothesis, these genes were not, or poorly, expressed in different TNBC cell lines, derived from either primary breast tumours or metastatic pleural effusions. This conclusion was further supported in vivo, as immuno-histochemical analysis on biopsies of TNBC invasive ductal carcinomas highlighted differential expression of these six genes in cancer cells, as well as in intra- and peri-tumoral infiltrating lymphocytes. Our data open to the possibility that inter-tumour heterogeneity of immune markers might have predictive value; further investigations are recommended in order to establish the real power of cancer-related immune profiles as prognostic factors.

**Supplementary Information:**

The online version contains supplementary material available at 10.1007/s12672-021-00401-0.

## Introduction

Breast cancer is the most commonly diagnosed cancer among women aged 20–60 years, with over 2,000,000 new diagnosed cases every year, worldwide. Although incidence rates have been stable or even decreased in recent years, nonetheless this tumour remains the second leading cause of cancer death in women [1, 2].

Difficulty in setting effective therapeutic treatments resides in the phenotypic heterogeneity observed within breast cancer subtypes. Integrated information across different molecular platforms (mRNA and protein expression, DNA methylation, microRNA and whole exome sequencing, SNP arrays) identifies four main breast cancer classes [namely luminal A, luminal B, human epidermal growth factor receptor 2 (HER2)-enriched and basal-like subtypes], differing in terms of prognosis and response to therapy [3, 4]. Luminal A and B breast cancers, characterized by positivity for estrogen receptor (ER), are the most heterogeneous ones, but are well responsive to hormone therapy, while patients carrying the HER2 subtype of breast cancer are sensitive to trastuzumab treatment [5]. The most aggressive and with the worst prognosis are the basal-like subtypes, also referred to as triple-negative breast cancers (TNBC), because more than 80% of these tumours are typically negative for ER, HER2 and progesterone receptor (PR); this phenotype makes TNBC hardly responsive to available and accessible therapies [5].

During the last few years, new evidence has highlighted the key role of host immune-surveillance in influencing tumour biology. Basically, in the early stages of cancerogenesis, tumour-associated antigens can prime immune cells [macrophages, natural killer (NK) cells and CD8^+^ cytotoxic T lymphocytes (CTL)] present in the stromal microenvironment, which, in turn, elicit a potent anti-tumour response [6, 7] that could also be manipulated for tissue repair [8]. The beneficial effects of immune surveillance are proven by the finding that levels of tumour infiltrating lymphocytes (TIL) strongly correlated with better prognosis in several cancer types, including melanoma, colorectal, oral squamous cell, ovarian and breast carcinomas [9], as well as in the microbiome [10, 11]. Over time, however, an “escape phase” is established, allowing transformed cells to survive, thus leading to tumour progression and invasion [12, 13]. Immune evasion strategies occur via multiple mechanisms, including down-regulation of tumour-associated antigens, increased expression of pro-survival/resistance genes, development of immune tolerance, and establishment of an immune-suppressive microenvironment [7, 14, 15].

Based on these findings, cancer immunotherapy is currently been developed, in order to enhance innate and adaptive immune responses [16, 17]: in particular, immune checkpoint inhibitors [like those of CTLA-4, programmed death receptor-1 (PD-1) and its ligand PDL-1] are among the most innovative approaches designed for antagonizing immune tolerance and inducing tumour regression [18–21].

Taken together, all these evidences underline the relevance of immune responses in cancer biology and suggest immune profiles as useful tools for improvement of diagnosis and prognosis in breast cancer. By a bioinformatic approach, we checked for potential prognostic factors in TNBC: our analysis showed that some immune-related genes were associated with disease-free survival and, therefore, may be promising factors for estimating the natural history of the tumour and the chance of disease recurring.

## Materials and methods

### Cell cultures

The breast cancer cell lines HCC1937, MDA-MB-231, MDA-MB-436, MDA-MB-453, MDA-MB-468, BT20 and BT549 were obtained from American Type Tissue Culture (Manassas, VA, USA). BT549 and HCC1937 cells were maintained in RPMI-1640 culture medium, while the other cell lines were maintained in Dulbecco's modified Eagle's medium (DMEM). Both media were supplemented with 10% FBS, 100 μg/mL kanamycin, 0.1 mg/mL sodium pyruvate (Biowest, Texas, USA) and cultured in a humidified 5% CO_2_ atmosphere at 37 °C.

### Real time polymerase chain reaction (RT-PCR)

Total RNA was extracted by using the RNeasy Mini Kit, according to the manufacturer’s protocol (Qiagen, Hilden, Germany). One μg of RNA was reverse transcribed by using the GoTaq Reverse Transcription System (Promega, Madison, WI, USA), and the resulting cDNA was amplified on a 7500 Fast Real-Time PCR system (Applied Biosystems, Thermo Fisher Scientific, Waltham, MA, USA), by using GoTaq qPCR Master Mix (Promega), with processing at 95 °C for 10 min, 95 °C for 15 s and 60 °C for 1 min, for 40 cycles. The relative expression levels of genes were calculated using the 2^−ΔΔCt^ method. RPL21 was used as reference gene in all reactions.

### Western blot

Cells were lysed in RIPA buffer (1% NP-40, 0.1% SDS, 150 mM NaCl, 50 mM Tris–HCl pH 7.5, 0.5% sodium deoxycholate), containing protease inhibitor cocktail (Sigma, Saint Louis, MO, USA). Twenty μg of total proteins were separated by SDS-PAGE, transferred onto PVDF membrane (GE Healthcare, Little Chalfont, UK), incubated with the specific antibodies and detected with enhanced chemiluminescence kit (Santa Cruz Biotechnology, Santa Cruz, CA, USA). The primary antibodies used were goat anti-TRIM 1:2000 (R&D System, Minneapolis, MN, USA), rabbit anti-CD53 1:1000 (Abcam, Cambridge, UK), rabbit anti-Jaw1 1:500 (Abcam), rabbit anti-IL18R1 1:250 (Novus Biologicals), mouse anti-LTB 1:500 (Abcam), rabbit anti-PTPRCAP 1:1000 (ProteinTech, Rosemont, IL, USA), rabbit anti-tubulin 1:500 (Santa Cruz Biotechnology).

### Bioinformatic analysis

Bioinformatic analysis was carried out utilizing the gene expression data sets Metabric [22] downloaded from the original portal or from the GEO omnibus repository (http://www.ncbi.nlm.nih.gov/geo/). The Metabric dataset [22, 23], with subsequent updates, contains expression profile data of over 2000 breast cancer subtypes (among them, more than 200 specimens represent TNBC) with over 10 years follow-up, allowing a solid statistical base and separation of the different breast cancer subtypes. The rank normalization was applied before analyses. Based on normalized expression values of gene of interest, samples were split into 2 groups, those with high expression (above median) and with low expression (below median). We used tool we developed and described previously [23–26], see also SynTarget [27], DRUGSURV [28] and p53MutaGene [29]. A further description of bioinformatics analyses and algorithms used in this study can be found in [30].

### Immunohistochemistry

The expression of IL18R1, LTB, PTPRCAP, CD53, Jaw1 and TRIM was evaluated, by immunohistochemistry, on 12 cases of invasive ductal TNBCs. The age of patients was between 42 and 60 years; five subjects experienced disease progression, while the remaining seven showed no progression. Concerning histological tumour characteristics, one case was pT1, ten were pT2 and one was pT4. Four cases showed the presence of lymph node metastases, while in the other samples lymph node negativity was found.

Briefly, antigen retrieval was performed on 3-μm-thick paraffin sections, by using EDTA-citrate pH 7.8 or citrate pH 6.0 buffers, for 30 min at 98 °C in a thermostatic bath, according to Additional file 2: Table S1. Sections were, then, incubated with primary antibodies (listed in Additional file 2: Table S1), at room temperature for different incubation times, depending on the antibody employed. After washings with TBS/Tween20 pH 7.6, antibody positivity was detected by HRP-DAB Detection Kit (Novolink Polymer Detection Systems, Leica Biosystems Newcastle Ltd, UK). Immunohistochemistry was evaluated by two blind observers (LA and AM), by counting the number of positive breast cells (% of positive breast cancers cells).

### Statistical analysis

Data are presented as means ± SD. Statistical analysis was performed by Student’s t-test. For survival analyses, R package (https://www.emilyzabor.com/tutorials/survival_analysis_in_r_tutorial.html) was used and the log-rank p-value was reported. P-values of ≤ 0.05 were considered statistically significant.

## Results

### Immune-related genes are associated with good prognosis in breast cancer

In order to investigate potential prognostic factors in breast cancer, we performed a bioinformatic analysis, by using the tool we developed and described previously [24–26], using the Metabric dataset [22], with subsequent updates, containing expression profile data of 2000 breast cancer specimens with over 10 years follow-up.

**Fig. 1:**
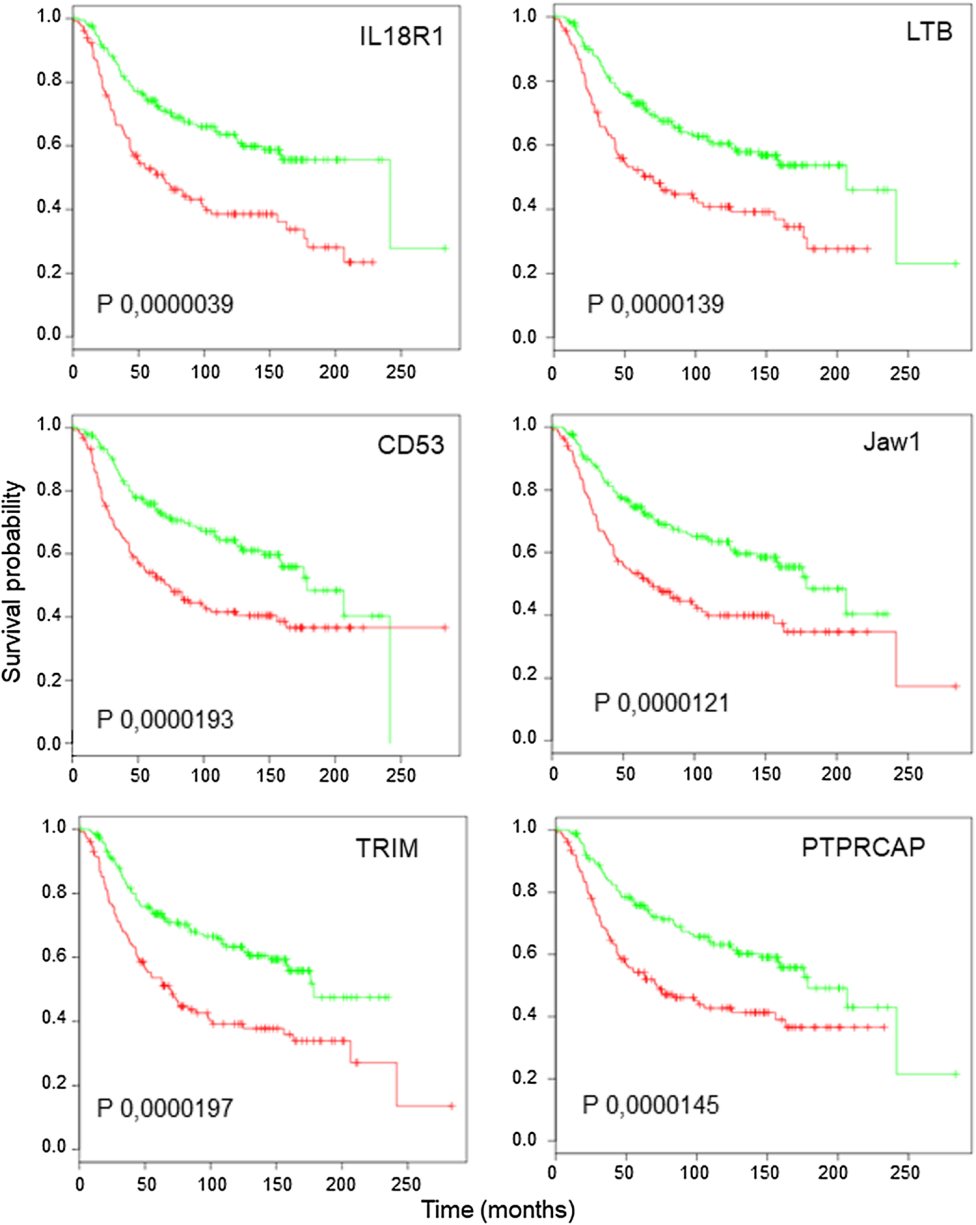
Kaplan–Meier plot based on the expression level of six mRNAs. Disease-free survival curves were estimated for high- (green lines) and low- (red lines) expression in TNBC patients. Datasets: Metabric, see main text. The p values are indicated in each panel

We identified several genes whose expression showed a strong positive correlation with disease-free survival (up to 20 years) in TNBC patients (Fig. 1 and Additional file 1: Fig. S1). Out these, we decided to perform further biochemical analysis on IL18R1, LTB, CD53, Jaw1, TRIM, PTPRCAP (Fig. 1). The rational was the statistical power, always above 10^–5^ (in fact, 0.0000039 for IL18R1).

Interestingly, most of these genes are mainly expressed in (although not restricted to) immune cells (including B- and T-lymphocytes, monocytes, neutrophils and platelets) and are required for productive lymphocyte activation. Among specific functions, these genes are involved in T cell receptor signalling [e.g*.*, T cell receptor associated transmembrane adaptor 1 (TRIM) and CD45-associated protein (or protein tyrosine phosphatase, receptor type, C-associated protein; PTPRCAP)], cytokine production and inflammatory response [e.g., interleukin 18 receptor 1 (IL18R1) and leukocyte surface antigen CD53 or tetraspanin 25 (CD53)], immune response regulation and normal development of lymphoid tissue (e.g., lymphotoxin β (LTB or TNF-C) and lymphoid-restricted membrane protein (Jaw1)].

Improved survival outcome related to high expression suggested that some of these genes may be promising biomarkers of life expectancy.

### Analysis of expression in TNBC cell lines

Unexpectedly, the best correlation with survival was not with genes expressed by cancer cells, but by infiltrating host cells, and in particular immune cells. Nonetheless, both mRNA differential expression (RNAseq, microarray, SAGE) and integrated proteomics (ProteomicsDB, MaxQB, and MOPED) reported the presence of these genes in cells other than immune cells [31–36]. Therefore, we tested in vitro the expression of these markers with a set of cancer cells, to formally support the observation that these were indeed host immune genes. This, in keeping with the concept of immune checkpoint blockade, where host immune regulators (such as CTLA4 and PDL1) show a dramatic effect on cancer progression (regardless of cancer genetic mutations or subtypes). To this end, we checked, at mRNA and protein levels, the expression of selected genes in a panel of TNBC cell lines, derived from either primary breast tumours (HCC1937, BT-20, BT-549) or metastatic pleural effusions (MDA-MB-231, MDA MB 436, MDA MB 453, MDA-MB-468).

**Fig. 2:**
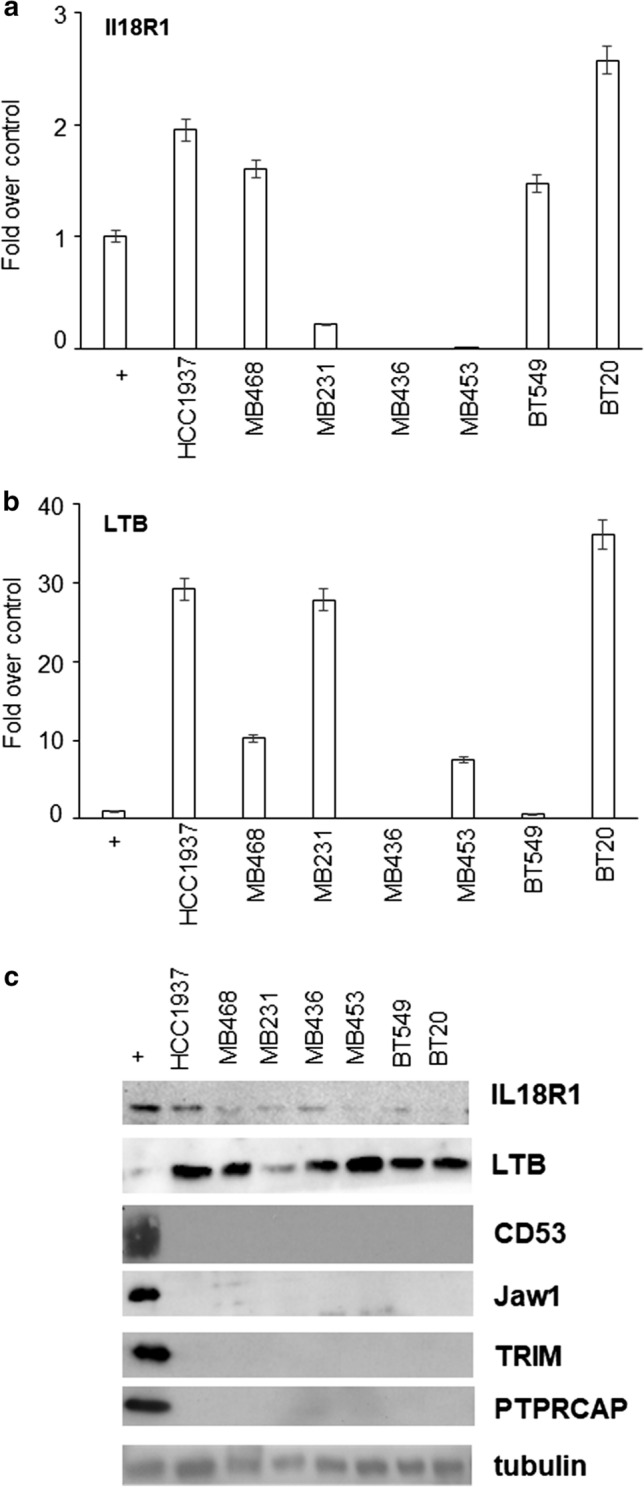
Expression of six mRNAs in breast cancer cell lines. Real-time PCR (**a** and **b**) and Western blot (**c**) were performed on the indicated TNBC cell lines. Positive controls (+) were extracts from Jurkat cells. Only graphs related to mRNA expression of IL18R1 (**a**) and LTB (**b**) are shown. Data are reported as fold over positive control (set to 1), after normalization to tubulin content (S.D. ≤ 5%). Blots are representative of three independent experiments

As expected, four genes out of six were not expressed in any of the tested cell lines, as assessed by real-time PCR (data not shown) and Western blot (Fig. 2) (Additional file 3). Conversely, the different cell lines showed variable expression of LTB and IL18R1 (Fig. 2), as already reported in different epithelial and carcinoma cells [32–34, 37]. In particular, almost all cell lines showed high LTB protein levels, except for MDA-MB-231 cells. Concerning IL18R1 expression, highest levels were seen in HCC1937 and MDA-MB-436 cells; of note, IL18R1 has been recognized as a differentially expressed gene in *BRCA1* mutation carriers and HCC1937 and MDA-MB-436 are the only two cell lines that harbour *BRCA1* gene mutation [38].

### Analysis of expression in breast cancer specimens

In order to confirm our data in vivo, we performed immune-histochemical analysis on biopsies, deriving from twelve patients with triple negative invasive ductal carcinoma (mean disease-free survival: 8.9 ± 1.6 years), almost all in pT2, with no or few tumour spread to the lymph nodes.

**Fig. 3:**
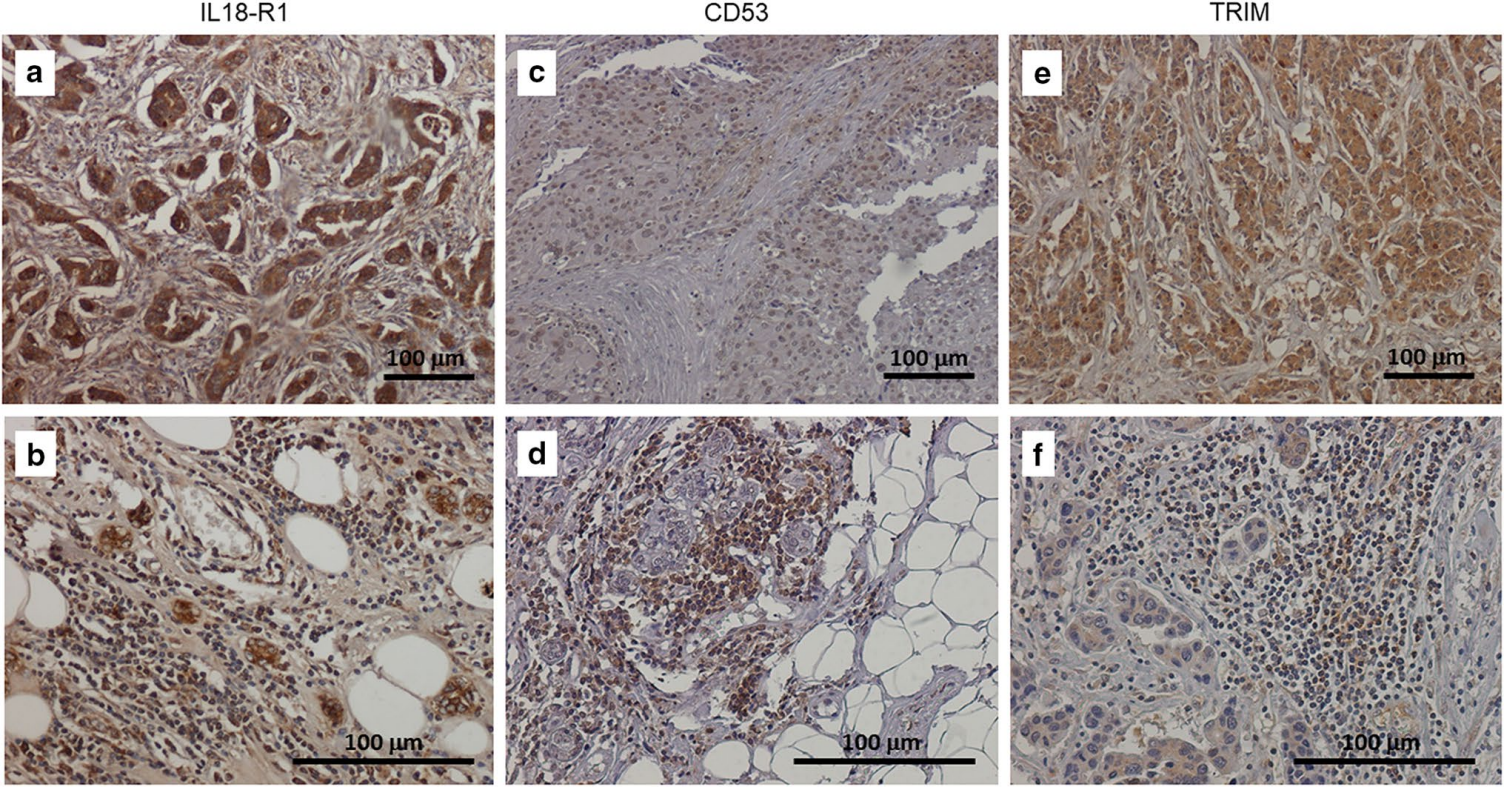
In situ evaluation of some prognostic markers in TNBC samples. **a** and **b** anti-IL18R1 staining. A marked cytoplasmic and membrane positivity was found in about 90% of breast cancer cells (Panel a) and 50% of intra-tumour lymphocytes (Panel b). **c** and **d** anti-CD53 staining. A moderate nuclear staining was observed in about 60% of neoplastic cells (Panel c) and 70% of intra-tumour lymphocytes (Panel d). **e** and **f** anti- TRIM Ab staining. A moderate cytoplasmic and nuclear positivity was present in about 70% of TNBC cells (Panel e) and 30% of intra-tumour lymphocytes (Panel f)

As shown in Table 1, variable positivity for all tested antibodies in cancer cells, as well as in intra- and peri-tumoral TILs, was found. CD53, absent in non-tumour ducts, was detected in 50% of breast carcinomas and, in the positive cases, a weak nuclear positivity was observed in 5–75% of neoplastic cells (Fig. 3c and d). Conversely, CD53 resulted highly expressed both in intra- and peri-tumoral lymphocytes (> 80% positive cells), in all specimens, where intense nuclear positivity was found (Fig. 3c and d). Similar expression pattern has been observed for the PTPRCAP antibody: only 41.7% of breast carcinoma cells expressed the protein, while TILs were all positive, in all analyzed samples. However, PTPRCAP showed expression variability greater than CD53: in the positive cases, nuclear and cytoplasmic staining was seen in a number of cells ranging from 5 to 100%.

**Table 1:** Summary of mRNA expression in breast cancer specimens

	Tumour cells	Non-tumour ducts	Intra-tumour TILs	Peri-tumour TILs
***IL18R1***	100% (12/12)	100% (12/12)	57.1% (4/7)	75% (6/8)
Positivity range	55–100%	100%	5–20%	5–50%
Intensity	++(+)	+++	++	+++
Localization	Cytosol/membrane			
***LTB***	25% (3/12)	0	Few	Few
Positivity range	15–60%			
Intensity	+			
Localization	Nucleus			
***CD53***	50% (6/12)	0	100% (7/7)	100% (10/10)
Positivity range	5–75%		80%	80–100%
Intensity	+(+)		++(+)	++(+)
Localization	Nucleus			
***Jaw1***	75% (9/12)	75% (9/12)	42.8% (3/7)	44.4% (4/9)
Positivity range	10–80%		40%	30–60%
Intensity	+	+(+)	+	+
Localization	Cytosol	Nucleus/cytosol	Cytosol	Cytosol
***TRIM***	91.7% (11/12)	50% (6/12)	71.4% (5/7)	70% (7/10)
Positivity range	15–80%		10–70%	7–70%
Intensity	+(+)	+(+)	+(+)	+(+)
Localization	Nucleus/cytosol	Nucleus/cytosol	Nucleus/cytosol	
***PTPRCAP***	41.7% (5/12)	0	100% (7/7)	100% (9/9)
Positivity range	5–70%		5–70%	10–100%
Intensity	+		++	++
Localization	Nucleus/cytosol

Nine out of twelve cases (75%) of breast cancer showed weak nuclear and cytoplasmic positivity for Jaw1 antibody, and the positivity range was 10–80%. The intra- and peri-tumoral inflammatory infiltrate resulted positive in about half of the cases (from 30 to 60% of lymphocytes).

IL18R1 and TRIM antibodies were positive in all tumour samples, although some variability was observed in number and staining intensity of positive cells. The percentage of neoplastic cells positive for both antibodies varied from 15 to 100%, with marked cytoplasmic and membrane positivity for IL18R1 antibody (Fig. 3a and b) and weaker nuclear and cytoplasmic positivity for TRIM antibody (Fig. 3e and f). Positivity for intra- and peri-tumour lymphocytes was also highly variable.

Finally, LTB was expressed only in 3 cases of breast cancer; it was expressed only in neoplastic cells (positivity percentage ranging from 15 to 60%), with non-tumour ducts and TILs always being negative.

Given the small number of cases examined, a significant correlation between the expression in situ of the various antibodies tested and the presence of lymph node metastases and disease recurrence was not found, even if it was not among the objectives of the study.

Interestingly, some of the investigated genes showed weak to intense nuclear positivity in breast specimens. Although we cannot rule out unspecific staining, nonetheless multiple databases and prediction tools, as well as available literature, described similar findings, as it is the case for Jaw1 and TRIM [39, 40]; in keeping, Jaw1 physically interacts with inner nuclear proteins and microtubules, thus contributing to maintenance of nuclear shape in a mouse melanoma cell line [39]. Currently, the functional role of these genes in the nucleus is still an open question and further studies might explore the biological significance of our immune-histochemical results.

## Discussion

Breast cancer is an heterogeneous disease, characterized by histopathological and genetically defined subtypes that make difficult to delineate specific prognostic factors and efficacious therapies [41], as it occurs also in several other tumours [42–44]. Indeed, the definition of prognostic markers is a serious issue [45, 46] that is a primary research goal for precision oncology [47, 48], highlighting the need for novel specific progression and prognostic molecular clusters able to stratify cohort of patients with distinct prognostic outcome [49, 50]. One of such markers, identified from the elucidation of novel molecular mechanisms underlying the progression of breast cancer, is the transcription marker p63. Like other cell death [51, 52] or redox regulators [53–57], TP63 [58–61], together with p73 [62–68], belongs to the p53 gene family and plays an essential role in development and homeostasis of stratified squamous epithelia and epithelial appendices, including breast [69, 70]. The TP63 gene is transcribed thanks to two distinct promoters, that results in generation of the isoforms TAp63 (containing the Trans-Activation domain, codified by exons 1–3) and ΔNp63 (Amino-Deleted isoform, lacking exons 1–3); moreover, both isoforms are able to undergo alternative splicing at the 3′-end, resulting in different variants, α, β, γ, δ and ε [71–73]. A crucial molecular determinant of the malignant behaviour of breast cancer, and in particular of the metastasizing capacity, is the p63 transcriptional target gene SHARP1 (known also as DEC2 or BHLHE41) that, in triple breast cancer, results in a very negative prognostic fate [74]. Interestingly, SHARP1 is regulated by p63 via the hypoxia-inducible factor 1α (HIF-1α) and HIF-2α, as p63 is able to physically bind HIF and promote its proteasomal degradation, independent of the von Hippel-Lindau tumour suppressor (pVHL) [74]. Interestingly, also the other p53-family member p73 is able of a similar mechanism [75]. In fact, p73 physically binds HIF1α and regulates proteosomal degradation of HIF, in a pVHL-independent fashion [76]. More, mutant p53 comes also into the equation, this time cooperating with HIF1α to transcribe novel genes, that foster cancer progression [77].

p63 promotes mammary stemness through the direct transcriptional control of the Frizzled 7 (FZD7) receptor and, at a lesser extent, of the WNT5B ligand [78]. FZD7 overexpression results in increased mammary stem cell activity and can rescue the impaired self-renewal of ∆Np63-depleted progenitors [78]. These observations imply that p63 enhances self-renewal of mammary stem cells by activating the WNT-β-catenin signalling pathway: the WNT ligand signal is conveyed to the cytoplasm through Dishevelled (DVL), which inhibits the function of a β-catenin destruction complex formed by axin, APC (adenomatous polyposis coli), and GSK-3β (glycogen synthase kinase-3β), thus leading to increased cytosolic β-catenin [69, 78]. At the same time, p63 contributes to the stemness phenotype through its ability to bind to promoters of several components of the Sonic Hedgehog (Hh) signalling pathway and its three ligands—Sonic (SHH), Desert, and Indian (IHH)—and to the Patched (PTCH) receptors that inhibit the G-coupled transmembrane protein Smoothened (SMO); in turn, GLI proteins become transcriptional activators (GLI^A^) and induce the expression of Hh target genes, including those involved in stem cell regulation (for example, *Bmi1*) [69, 79]. In this manner, p63 regulates the stemness compartment in breast cancer [69], while inhibiting cell death [80]—a process regulated by p63-triggered proteasomal degradation [81–84]. Additional mechanisms involving p63 and tumour progression have been evoked in breast cancer [85, 86], as well as for other tumours [87, 88].

Here, we looked for novel molecular signatures for TNBC, not directly related to cancer cells, but rather correlated with immune cells penetrating the tumour microenvironment. We relied on bioinformatic evaluation of the Metabric portal. This dataset, with 2000 samples and over 10 years follow-up eliminates the major study limitation consisting in the small sample size, allowing novel biomarkers to emerge and be validated. Among them, LTB resulted completely absent in non-tumour ducts, while variable expression was found in cancer epithelial cells. Interestingly, in primary melanoma and breast carcinomas, LTB has been correlated with high density of tumour-associated high-endothelial venules (HEVs), mediating the extravasation of T lymphocytes; consequently, elevated TIL levels in the breast tumour microenvironment lead to tumour regression [89]. In keeping, loss of tumour HEVs and LTB expression seem to be critical steps during breast cancer progression [90]. Therefore, LTB expression levels might be associated with favourable clinical outcomes. Similar variability has been found for IL18R1, expressed in both normal and transformed cells, as well as in intra- and peri-tumoral lymphocytes. Conflicting results (either pro-tumorigenic or suppressive roles) have been reported for IL-18 signalling in tumour development and progression. These discrepancies may be explained bearing in mind that IL-18 may recruit, beside the R1 receptor (α chain), also the R2 receptor (β chain), thus potentially activating different signalling pathways [91]. In addition, IL18R1 binds the anti-inflammatory and immune-suppressive IL-37 [92]; despite dampening host's immune responses, this interleukin shows interesting anti-tumour properties [93] and, in oral squamous cell carcinoma, serum IL-18/IL-37 ratio seems to regulate CD19^+^ B cells and CD3^+^ CD8^+^ T cells, thus representing a biomarker for predicting overall survival and disease-free survival [94]. Intriguingly, IL18R1 deletion increased tumour growth and burden, in mouse models of liver tumorigenesis, and the analysis of hepatocellular carcinoma patients indicated that IL18R1 exerted tumour-suppressive effects, largely by modulating activity of both CD8^+^ and multiple subsets of CD4^+^ T-cells; moreover, differences in expression levels in tumor tissue versus matched non-tumour tissue was more predictive of patient outcome than overall tissue expression [95].

Among genes showing high positivity in both intra- and peri-tumour TILs, CD53 appears to be a good candidate as prognostic marker for cancer patients, since it plays a key role in anti-tumour immunity, as already reported for other members of the tetraspanin superfamily [96]. The tetraspanin “web” regulates protein trafficking and signalling, as well as cell-to-cell adhesion and migration [97]; therefore, tetraspanins (including CD53) influence both T and B cell proliferation, as well as leukocyte migration into tumour microenvironment [98–102]. In this context, it is noteworthy that CD53, whose expression is mainly restricted to hematopoietic cells (including B and T cells, dendritic cells, NK cells, granulocytes, monocytes/macrophages), has been recognized as a regulator of IL-6 and IL-1β; in addition, genome-wide linkage studies have revealed an association between CD53 and innate TNF-α levels [103, 104]. CD53 is also involved in tumour antigen uptake by dendritic cells (DCs), the most professional antigen-presenting cells that activate and direct T cells toward the tumour, thereby promoting tumour immune-surveillance. This hypothesis is corroborated by the finding that CD53 (i) is highly expressed in all DC subsets (in particular, in plasmacytoid DCs) [105] and (ii) interacts with MHC class I molecules, thus functioning in cross-presentation [106]. The suggested predictive value of CD53 is supported by the finding that CD53^−/−^ mice showed increased tumour growth with respect to wild type counterparts, by using syngeneic immunogenic tumour models [96] and, furthermore, CD53 network predicts distant metastasis-free survival, especially in ER^−^ breast cancer [107].

Given the relevance of stromal components and immune response in recognizing and countering tumour cells, our data open to the possibility that inter-tumour heterogeneity of inflammatory markers may play a fundamental role as prognostic factor. The real predictive value of these markers will be established only after multiple, long-term, high-quality studies, but this pioneering work lays the foundation for future research.

## Supplementary Information

Below is the link to the electronic supplementary material.

**Additional file 1: Fig. S1.** Kaplan–Meier plot based on the expression level of three mRNAs not considered for further analyses. Disease-free survival curves were estimated for high- (green lines) and low- (red lines) expression in TNBC patients. Metabric dataset, see main text. The p values are indicated in each panel.

**Additional file 2: Table S1.** Immunohistochemistry conditions for expression analysis on breast cancer specimens.

**Additional file 3.**

## Data Availability

Not applicable. Not applicable.
