# Supplementary figures and images for: New immunological potential markers for triple negative breast cancer: IL18R1, CD53, TRIM, Jaw1, LTB, PTPRCAP
Source: Discov Oncol. 2021 Mar 10;12:6. doi: 10.1007/s12672-021-00401-0 (PMC8777524; doi:10.1007/s12672-021-00401-0)

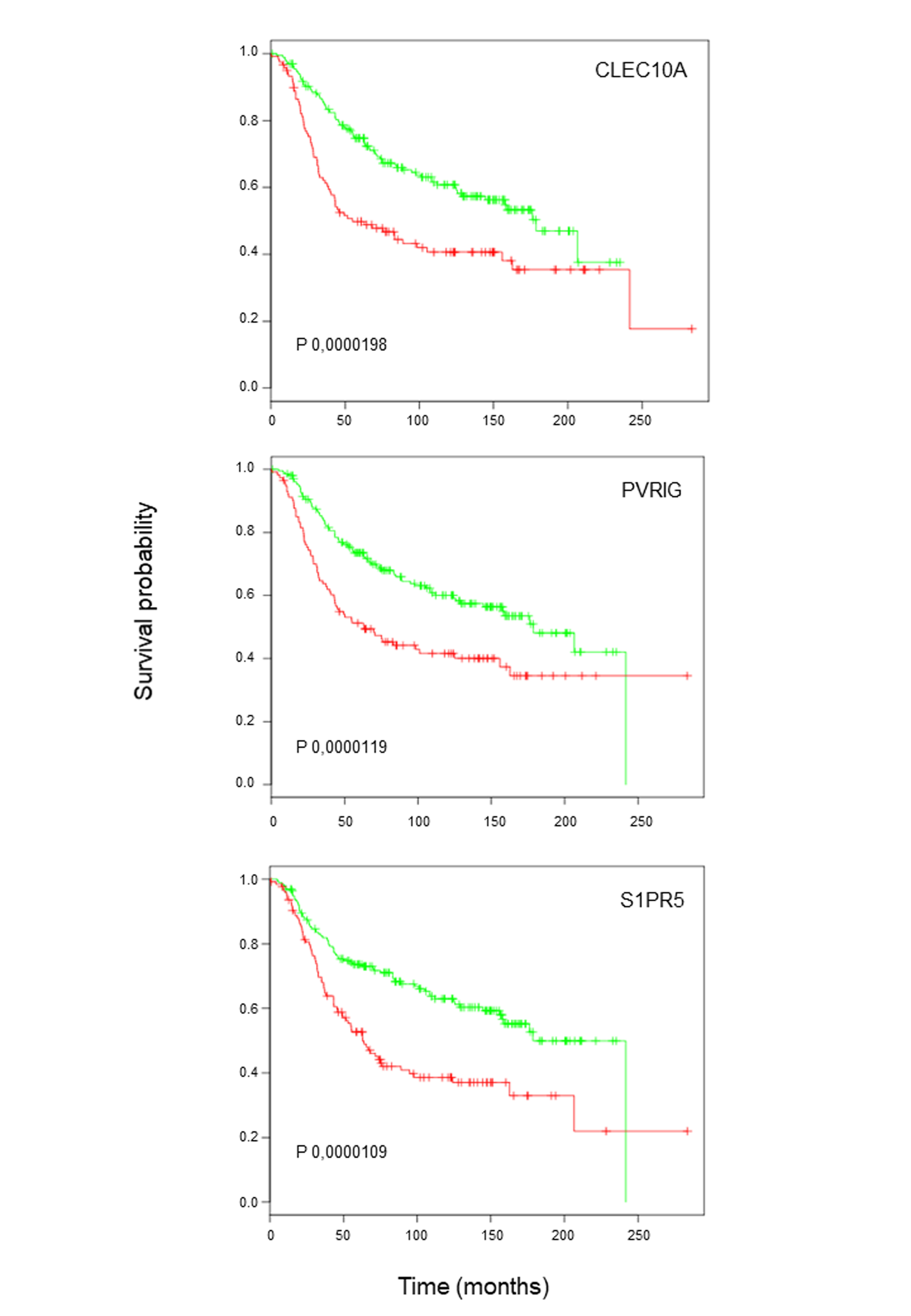

Supplement: Supplementary file 1 — Additional file 1: Fig. S1. Kaplan–Meier plot based on the expression level of three mRNAs not considered for further analyses. Disease-free survival curves were estimated for high- (green lines) and low- (red lines) expression in TNBC patients. Metabric dataset, see main text. The p values are indicated in each panel. [file 12672_2021_401_MOESM1_ESM.tif]

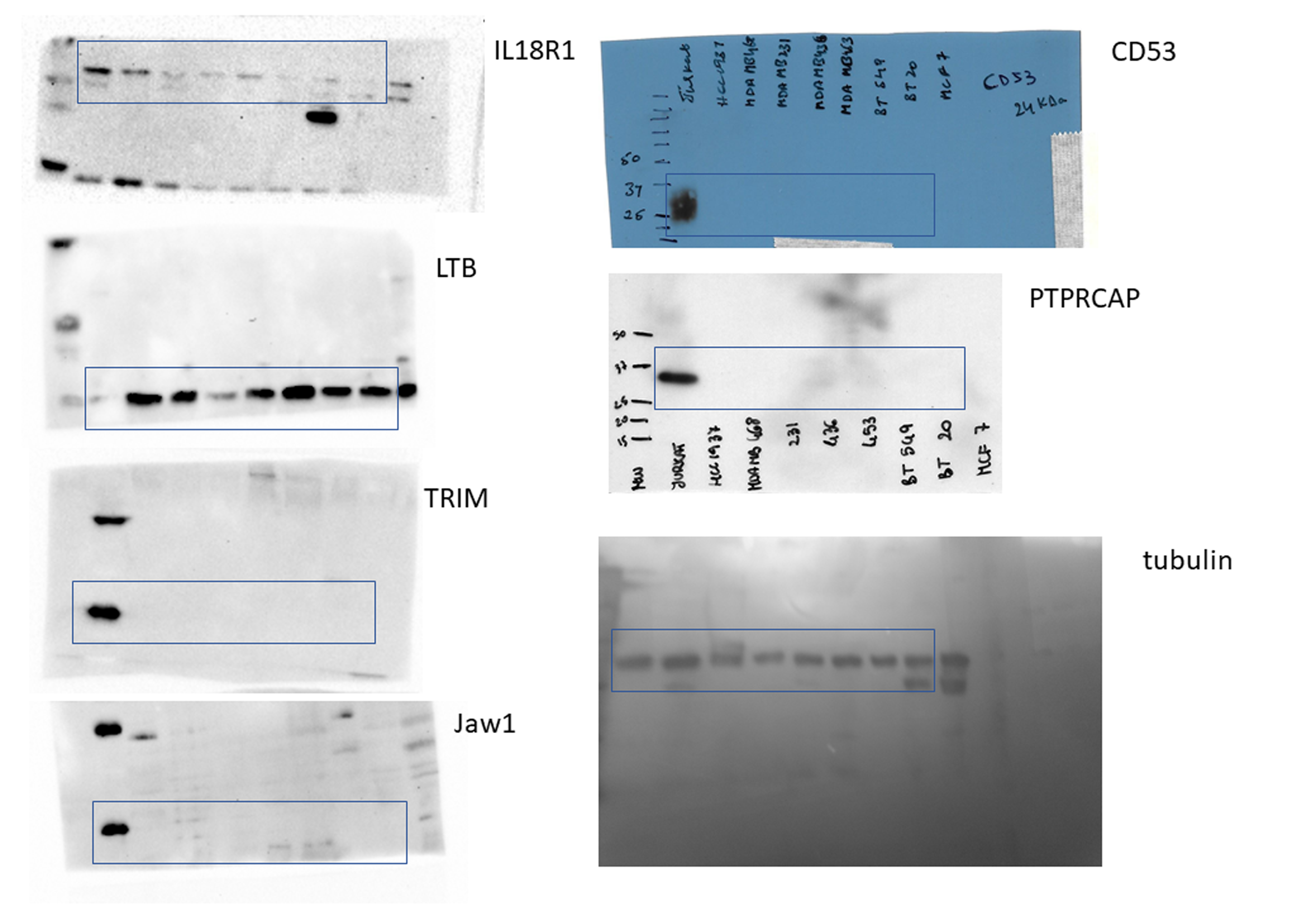

Supplement: Supplementary file 3 — Additional file 3. [file 12672_2021_401_MOESM3_ESM.tif]
