# Supplementary material for: New immunological potential markers for triple negative breast cancer: IL18R1, CD53, TRIM, Jaw1, LTB, PTPRCAP
Source: Discov Oncol. 2021 Mar 10;12:6. doi: 10.1007/s12672-021-00401-0 (PMC8777524; doi:10.1007/s12672-021-00401-0)
Supplement: Supplementary file 2 — Additional file 2: Table S1. Immunohistochemistry conditions for expression analysis on breast cancer specimens. [file 12672_2021_401_MOESM2_ESM.docx]

**Table S1.**

**Immunohistochemistry conditions for expression analysis on breast cancer specimens**

| **Antibody** | **Characteristics** | **Reaction target** | **Dilution** | **Retrieval** | **Time** |
| --- | --- | --- | --- | --- | --- |
| **IL18-R1** | Rabbit polyclonal clone NBP1-85782 (Novus Biologicals) | Antigen sequence:  HNGKLFNITKTFNITIVEDRSNIVPVLLGPKLNHVAVELGKNVRLNCSALLNEEDVIYWMFGEENGSDPNIHEEKEMRIMTPEGKWHASKVLRIENIGESNLNVLYNCTVASTGGTDTKSFILVRKA | 1:25 | EDTA citrate pH 7.8 | overnight |
| **LTB** | Rabbit polyclonal clone HPA048884 (Atlas Antibodies, Bromma, Sweden) | Antigen sequence:  GGLVTETADPGAQAQQGLGFQKLPEEEPETDLSPGLPAAHLIGAPLKGQGLGWETTKEQAFLTSGTQFSD | 1:100 | Citrate pH 6.0 | 1 hour |
| **CD53** | Rabbit monoclonal clone ab-134094 (AbCam) | Synthetic peptide corresponding to residues on the C terminus of human CD53 | 1:100 | EDTA citrate pH 7.8 | 1 hour |
| **Jaw1** | Rabbit polyclonal clone ab-88318 (AbCam) | Synthetic peptide at the C terminus of human Jaw1 | 1:100 | EDTA citrate pH 7.8 | 20 minutes |
| **TRIM** | Rabbit polyclonal clone NBP1-89660 (Novus Biologicals) | Antigen sequence:  RQDKMYSYSSDHTRVDEYYIEDTPIYGNLDDMISEPMDENCYEQMKARPEKSVNKMQEATPSAQATNETQMCYASLDHSVKGKRRKPRKQNTHFSDKDGDEQLHAI | 1:100 | Citrate pH 6.0 | 1 hour |
| **PTPRCAP** | Mouse monoclonal clone (17A5) sc-59290 (Santa Cruz Biotechnology) | Antigen sequence: cytoplasmatic domain of human PTPRCAP | 1:100 | Citrate pH 6.0 | 1 hour |
